# The Immune Response to Astrovirus Infection

**DOI:** 10.3390/v9010001

**Published:** 2016-12-30

**Authors:** Shauna A. Marvin

**Affiliations:** Biology Department, Drake University, Des Moines, IA 50311, USA; shauna.marvin@drake.edu; Tel.: +1-515-271-2811

**Keywords:** astrovirus infection, astrovirus replication, immune response, innate immunity, adaptive immunity, type I interferon

## Abstract

Astroviruses are one of the leading causes of pediatric gastroenteritis worldwide and are clinically importantly pathogens in the elderly and immunocompromised populations. Although the use of cell culture systems and small animal models have enhanced our understanding of astrovirus infection and pathogenesis, little is known about the immune response to astrovirus infection. Studies from humans and animals suggest that adaptive immunity is important in restricting classic and novel astrovirus infections, while studies from animal models and cell culture systems suggest that an innate immune system plays a role in limiting astrovirus replication. The relative contribution of each arm of the immune system in restricting astrovirus infection remains unknown. This review summarizes our current understanding of the immune response to astrovirus infection and highlights some of the key questions that stem from these studies. A full understanding of the immune response to astrovirus infection is required to be able to treat and control astrovirus-induced gastroenteritis.

## 1. Introduction

Astroviruses (AstV) are small, nonenveloped, RNA viruses that are a major cause of gastroenteritis in infants, immunocompromised people, and the elderly, and they also cause disease in mammals and birds [1,2,3,4,5,6,7,8,9]. Despite the disease burden, little is known about the immune response to astrovirus infection. Human clinical studies have demonstrated that an antibody-mediated response may be responsible for limiting astrovirus infection and clinical disease. Recent work using small animal models and cell culture systems have revealed an important role in the innate immune response in restricting astrovirus replication and pathogenesis. This review will summarize the current knowledge of the innate and adaptive immune responses to astrovirus infection using studies of humans, small animal models, and cell culture systems and will discuss how astroviruses evade the immune system. This review will also highlight the increasing reports of astroviruses as possible causes of central nervous system disease, especially in immunocompromised individuals. Finally, we will conclude with unanswered questions, future studies, and how the use of a newly developed mouse model can enhance our understanding of the immune response to astrovirus infection, and how these responses play a role in astrovirus-induced disease. 

## 2. Astrovirus Infection 

Astrovirus infection begins by binding to an unidentified receptor(s) on epithelial cells in the intestine after fecal-oral transmission [10,11] and enters cells via clathrin-mediated endocytosis [12]. After acidification of the endosome, endosome rupture, [12] and viral uncoating, two nonstructural proteins are translated from the single stranded, positive-sense genomic RNA from open reading frames (ORFs) 1a and 1b [13,14]. These polyproteins are cleaved into the non-structural proteins required for transcription and viral replication. Negative-strand RNA is produced from the genomic strand, and transcription of the negative-strand yields the genomic and subgenomic RNA [15]. The structural proteins encoded in the third ORF (ORF2) are expressed from the subgenomic RNA [13,14]. New astrovirus particles have been observed on double membranes likely serving as the site for replication and assembly [16,17,18,19,20]. After assembly, the progeny virions exit the cell through a non-lytic mechanism promoted by caspase activation [21,22]. 

Although astroviruses are considered gastrointestinal pathogens, viral RNA and infectious viral particles have been recovered from extraintestinal organs in both animals and humans (Table 1). In the current small animal model, turkey poults, turkey astrovirus type 2 (TAstV2) was found in bursa, thymus, spleen, kidney, liver, skeletal muscle, marrow, pancreas and plasma by reverse transcription polymerase chain reaction (RT-PCR), immunofluorescence, and virus isolation [6]. However, these tissues were negative by in situ hybridization using a riboprobe to the capsid gene, suggesting that the virus only replicates in the intestines [6,23]. Whether astrovirus localization to these extra-intestinal tissues leads to pathogenesis remains to be directly determined, and astroviruses have been implicated as a cause of neurological and other diseases. Examples in animals implicate astroviruses as the cause of hepatitis in ducks, causing encephalomyelitis in minks [24,25,26] and encephalitis in cattle [27,28,29]. Identification of astroviruses found in extra-intestinal tissues in humans will be discussed later in this review.

## 3. Adaptive Immunity

The innate immune response provides signals to recruit the adaptive immune response, which controls viral infection at later times during an infection. This response is pathogen specific and has a memory component that induces a more rapid and robust response following a second infection with the same pathogen. The adaptive immune response contains two arms: the humoral, or antibody-mediated/B cell response, and the cell-mediated response, which involves antigen-specific cytotoxic T cells. Although the findings between the current turkey model and the newly emerging mouse model yielded conflicting results, the reports showing the biphasic age distribution as well as immunocompromised individuals have formed a general conclusion that the adaptive immune system is a major component in controlling astrovirus disease (reviewed in [39]). 

Early studies in the turkey poult model found little adaptive immune responses after TAstV-2 infection [40]. Koci et al. found very low levels of IgG in the serum and IgA in the bile 21 dpi [40]. Additionally, the CD4 to CD8 T cell ratios did not change at 5, 9, and 16 dpi when comparing TAstV-2 infected poults to uninfected poults [40], demonstrating that TAstV-2 infection is a poor inducer of adaptive immune responses. 

Studies using the newly emerging mouse model and clinical studies in humans demonstrate that the adaptive immune response is key in controlling astrovirus infection and disease. Experimental infection of recombination activating gene 1 (*rag1*) gene knockout mice (Rag1^−/−^), which lack mature B cells or T cells [41], had higher (2 logs) levels of murine astrovirus (MuAstV) RNA shed in their feces compared to wild type mice at 14 dpi [42]. The kidneys, liver, and mesenteric lymph nodes were positive for astrovirus RNA, whereas infected wild type mice were negative for astrovirus RNA in these organs [42]. Rag1^−/−^ mice also contained 3–4 logs higher astrovirus RNA levels in the intestines compared to wild type controls [42]. These data indicate that the adaptive immune response is important in controlling astrovirus infection. Similar to the studies with interferon (IFN)-α receptor knockout (IFNaR^−/−^) mice by Marvin et al., the Rag1^−/−^ mice were positive for astrovirus prior to experimental inoculation [42]. Therefore, a complete understanding in the adaptive immune response, and how it limits astrovirus replication, cannot be determined until MuAstV-negative Rag1^−/−^ mice are generated. 

Studies in immunocompetent humans also show that 70% of healthy adults have antibodies against astroviruses [43], indicating that an adaptive immune response is mounted in humans. In earlier clinical studies using human volunteers, the majority of subjects that had no or only mild clinical signs had developed anti-astrovirus antibodies [5,44]. Conversely, subjects that exhibited more severe disease did not develop anti-astrovirus antibodies [44]. Other recent studies have found astrovirus antibodies against astroviruses of other animal species in the absence of disease [45,46]. For example, humans that work closely with turkeys develop antibodies to TAstV [45].

Antibodies that recognize the spike domains on the human astrovirus spike, the domain of the capsid involved in astrovirus binding [47], and neutralized virus activity have been identified [48,49]. However, the development of anti-HAstV therapies has been hampered by the gap in knowledge of neutralizing antibodies epitopes on HAstV surfaces. Bogdanoff et al. mapped the neutralizing epitopes on the HAstV-2 with a neutralizing monoclonal antibody to the spike domain, which prevented spike binding to Caco2 cells [47]. The solving of the crystal structures of HAstV-1 and HAstV-8 capsids and spike domains, and the HAstV-2 and TAstV-2 spike domains can advance our understanding of anti-astrovirus antibody binding [47,50,51,52,53]. These structures are crucial for future studies to develop vaccines and antibody therapy prevention and treatment of astrovirus disease.

Increasing reports of astrovirus infection in extra-intestinal tissues in immunocompromised patients highlight the role of the adaptive immune response during astrovirus infection. Several astrovirus isolates have been identified as potential causes of encephalitis in immunocompromised human patients (Table 1). For example, astrovirus RNA was found in the central nervous system of a child with X-linked agammaglobulinemia who died of encephalitis [35], and have been thought to be the cause of encephalopathy after bone marrow or hematopoietic stem cell transplantation (HSCT) [36,37]. Astrovirus has also been detected in nasopharyngeal secretions and serum and plasma of severe immunodeficiency (SCID) patients after HSCT [32], and detection of astrovirus in the blood has been associated with fever [33]. As the number of reports of astrovirus detection in extra-intestinal tissues increase, how the immune systems limits spread of astrovirus to extra-intestinal tissue will be an important question for future studies.

Few studies have demonstrated a role for T cell-mediated control of astrovirus infection. Molberg et al. recovered astrovirus-specific CD4+ and CD8+ T cells from astrovirus-stimulated biopsies taken from the duodena of patients that had histologically normal intestines [54]. Wood et al. found chronic rotavirus and astrovirus infection in two children with T cell immunodeficiency [55]. Considering the fact that CD4+ T cells are essential in B cell maturation and antibody specificity; it is reasonable to suspect that T cells play a role in the immune response to astrovirus infection. 

## 4. Innate Immune Responses

**Figure 1:**
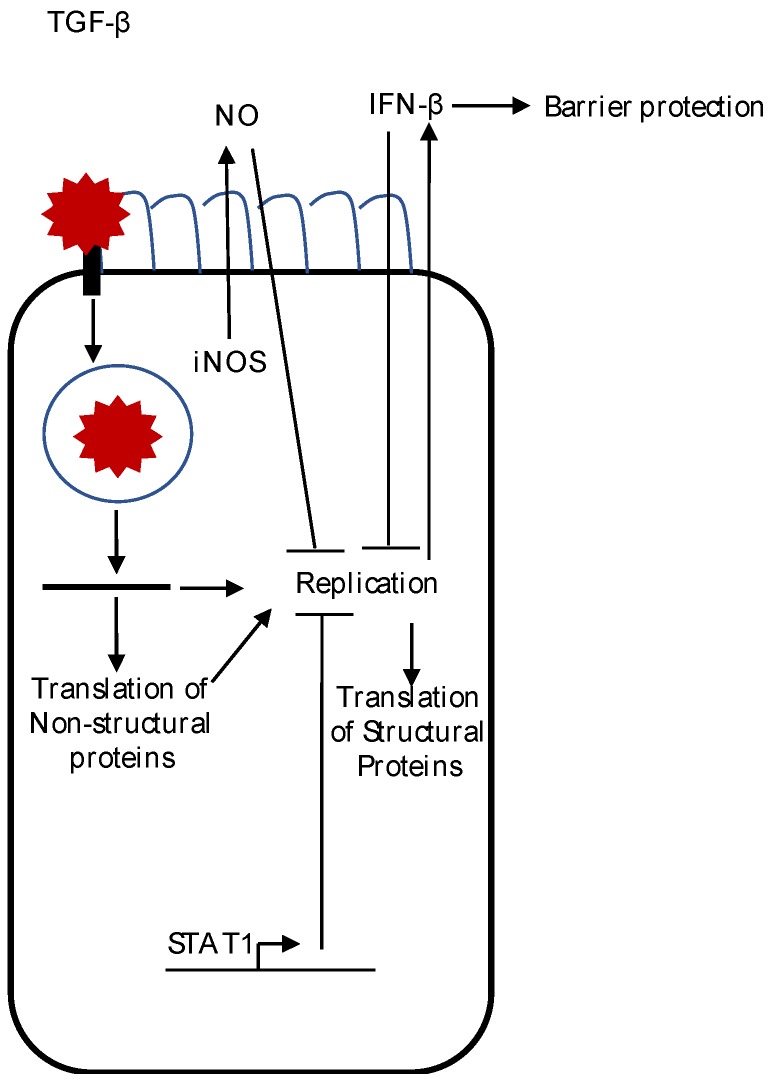
A summary of the innate immune response to astrovirus infection. After binding and entry into the cell, viral uncoating of the astrovirus genome occurs. Replication induces IFN-β production, which suppresses viral replication and translation of the structural protein, and decreases astrovirus-induced barrier permeability. Synthesis of inducible nitric oxide synthase (iNOS) protein also occurs during astrovirus infection of epithelial cells. Although replication is not needed to induce iNOS in macrophages, whether or not iNOS induction occurs during astrovirus infection of epithelial cells is unknown. Active transforming growth factor beta (TGF-β) levels are increased after astrovirus infection, but the role of TGF-β after astrovirus infection is not known. The presence of signal transducer and activator of transcription 1 (STAT1) decreases astrovirus replication, but the mechanism(s) remain to be determined.

The innate immune system is the first line of defense against an invading pathogen. In vitro studies in cell culture models and in vivo animal models implicate that innate immune responses may be important in controlling astrovirus infection and replication. Although knowledge of the innate immune response to astrovirus infection is still in its infancy, we will discuss the known responses, or lack of, including histological changes, nitric oxide production, and type I IFN production in astrovirus replication and pathogenesis (summarized in Figure 1).

### 4.1. Histological Changes

Unlike other enteric pathogens, such as rotavirus and enteropathogenic *Escherichia coli*, which cause inflammation and cell death [56,57], astrovirus infection causes only mild histological changes. Although turkey poults infected with TAstV2 had watery diarrhea by 24 h post infection (hpi), this diarrhea was not associated with inflammation and pathology, as few lesions and little cell death were observed in the intestinal villi [4,6]. In gnotobiotic lambs and calves infected with ovine astrovirus or bovine astrovirus, only mild destruction of the villi was observed with few observations of dying enterocytes [19,58]. These animal results are similar to what is seen in humans, with little destruction of the intestinal villi after astrovirus infection [59]. 

### 4.2. Nitric Oxide Production

Nitric oxide is a key mediator of the innate immune system and is involved in pathogenesis and the control of infectious pathogens. Studies by Koci et al. demonstrate that TAstV-2 infection stimulates nitrite production, which limits viral replication [40]. Splenocytes from infected turkey poults made more nitrite after lipopolysaccharide (LPS) stimulation after splenocytes from uninfected poults [40]. TAstV infection increased nitrite levels after in vivo infection [40] in isolated avian macrophages and a chicken macrophage cell line (HD11) [40,60]. Treatment with nitric oxide synthase (NOS) inhibitors increased TAstV-2 replication in vitro and in vivo, demonstrating for the first time a role in the innate immune system in controlling astrovirus infection [40]. Importantly, turkey inducible nitric oxide synthase (iNOS) was shown to be turned on in intestinal epithelial cells where astrovirus replication was occurring, suggesting that these cells are capable of mounting their own defense against astrovirus infection [61].

### 4.3. Type I IFN

Two recent, independent studies explored the type I IFN response during human astrovirus infection, providing the most comprehensive knowledge about an innate immune response to astrovirus infection to date [62,63]. Although each study used different astrovirus serotypes, both Marvin et al. (using HAstV-1) and Guix et al. (using HAstV-4) found that infection induced a small, but significant increase in IFN-β mRNA and protein levels at 24 hpi [62,63]. This increase in IFN-β mRNA levels was not seen after infection with either UV inactivation of the virus or HAstV1 recombinant capsid, indicating that this type I IFN response is dependent on viral replication [62,63]. The transcription factor IFN regulatory factor (IRF)-3, which, when phosphorylated, translocates into the nucleus to promote transcription of the IFN-β promoter, was located in the cytoplasm in infected cells at 12 hpi but was localized in the nucleus at 24–48 hpi [63]. Many RNA viruses, such as coronaviruses and rotaviruses, encode IFN antagonists [64,65,66,67]. However, Guix et al. found that HAstV-4 infection did not block polyinosinic:polycytidylic acid (polyI:C)-induced type I IFN levels, suggesting that HAstV-4 does not encode a protein that exhibits interferon–antagonist activity [63].

To determine if type I IFN limited astrovirus infection and replication, both groups pretreated the human intestinal adenocarcinoma cell line Caco2 with recombinant type I IFN prior to astrovirus infection. Guix et al. pretreated with a single dose of IFN alpha (IFN-α) and saw a signification reduction in new capsid protein synthesis and mRNA levels at all HAstV-4 multiplicity of infection (MOIs) used [63]. They also saw a 2-fold increase in viral RNA and infectious virion levels when inhibiting TANK-binding kinase 1 (TBK1), which lies upstream of IRF-3 [63]. Marvin et al. pretreated Caco2 cells with increasing amounts of IFN-β and saw a dose-dependent reduction in new capsid protein synthesis, and, importantly, found that the amount of IFN-β produced during astrovirus infection is sufficient to decrease infectious progeny virion levels, as treatment with an IFN-β neutralizing antibody during infection increased HAstV-1 titers by 2.5 log-units [62]. Marvin et al. also saw decreased levels of positive-strand RNA levels at 8 and 16 hpi in Caco2 cells pretreated with IFN-β [62]. This decrease was not observed at early time points (1 and 4 hpi), suggesting that IFN-β pretreatment does not decrease HAstV-1 entry [62].

In addition to limiting viral replication, both studies asked if type I IFN influences astrovirus-induced barrier permeability. Astrovirus infection has been shown to increase epithelial permeability, which correlated with the disruption of tight junction proteins and sodium malabsorption [3,4,68]. Guix et al. concluded a positive correlation between IFN-β RNA production and barrier permeability during HAstV-4 infection [63]. Marvin et al. demonstrated that type I IFN may be important in protecting the epithelial barrier during HAstV-1 infection. Pretreatment of Caco2 cells with IFN-β reduced the HAstV-1-induced decrease in transepithelial electrical resistance (TER) and decreased the transmigration of an inert dye (flux) from the apical side of the cell monolayer to the basolateral side [62]. Additionally, HAstV-1 infection in the presence of an IFN-β neutralizing antibody saw increased levels of flux compared to isotype control [62]. Although the permeability studies were performed in the absence of trypsin, and therefore only one round of replication occurred, it is possible that the varying levels of unprocessed viral particles (lower levels with IFN-β pretreatment and higher levels in the presence of an IFN-β neutralizing antibody compared to control cells) could contribute to the differences in barrier permeability since astrovirus-induced barrier permeability is independent of viral replication [68].

The effects of type I IFN signaling in astrovirus replication, clearance, and barrier permeability have been examined in vivo using IFNaR^-/-^ knockout mice. Oral gavage of wild-type C57BL/6 mice with MuAstV-positive fecal filtrates results in productive infection, viral shedding, and increased intestinal permeability [62]. However, the IFNaR^−/−^ mice used in the study were found to already be MuAstV-positive in their colony. The viral titers shed in the stool of wild-type mice peaked at 6 to 11 dpi, shedding similar levels as the IFNaR^−/−^ mice. However, wild-type mice titers rapidly decreased after 11 dpi and cleared the infection by 53 dpi, compared to IFNaR^−/−^ mice that shed virus at high levels (>10^6^ copies/μg RNA) at 53 dpi. These data suggest that type I IFN signaling is important for viral clearance in vivo [62]. IFNaR^−/−^ mice also had increased intestinal permeability compared to MuAstV-infected wild-type mice, which correlated with their in vitro observations, suggesting a role for type I IFN in protecting the epithelial barrier during infection, although MuAstV-free IFNaR*^−/−^* mice would need to be generated to definitively determine the role of type I IFN signaling in intestinal permeability [62].

Overall, the studies of Guix et al. and Marvin et al. demonstrate that the type I IFN system can limit astrovirus replication in vitro and in vivo and protects against astrovirus-induced barrier permeability. How astrovirus replication induces type I IFN production, and why induction is only seen late in infection and at low levels remains unknown. Additionally, how type I IFN protects against astrovirus-induced barrier permeability remains to be resolved. 

### 4.4. Additional Innate Immune Responses

Several observations in animals have described other innate immune responses during astrovirus infection. Astroviruses have been found in macrophages of lambs and described in calves with cells around Peyer’s patches [58,69]. However, whether these cells are activated in response to astrovirus infection have yet to be determined. TAstV-2 infection induces active TGF-β, an immunosuppressive cytokine that may explain why there are limited histological changes after infection [70], in the serum of infected turkey poults [6]. Whether TGF-β levels play a role in astrovirus replication and/or suppression of the immune response has yet to be determined.

Finally, a study in mice found a role for the signal transducer and activator of transcription 1 (STAT1) transcription factor, a factor known for controlling viral infection [71], in limiting MuAstV replication [42]. STAT1 knockout mice (STAT1^−/−^) shed 10-fold more viral copies in the feces 14 dpi compared to wild-type mice and had viral RNA in the spleen and the mesenteric lymph nodes, while viral RNA was not detected in those organs of wild-type mice [42]. Like type I IFN signaling, STAT1 has a role in limiting mouse astrovirus infection, but the mechanism(s) remains unknown. It is possible that type I IFN signaling leads to STAT1-mediated transcription of antiviral proteins that can inhibit astrovirus replication.

## 5. Astrovirus Evasion and Suppression of the Immune Response

Multiple studies have concluded that astrovirus may evade the immune system by preventing complement activation [72,73,74,75]. The complement system is a fundamental component of the immune response that detects pathogens through one of three pathways, the classical, the mannose-binding lectin (MBL) and the alternative pathways. Once activated, it can eliminate pathogens, regulate the inflammatory response, and help shape the adaptive immune response. The HAstV-1 capsid protein inhibits the classical and lectin pathways by binding to key initiator molecules C1q and MBL, preventing subsequent downstream activation [73,74,75]. Considering a critical role of complement is initiating an inflammatory response and the observations that astroviruses do not cause inflammation highly suggests that astrovirus inhibition of complement activation is a key player in evading the immune system and suppressing the inflammatory response. Indeed, Tam et al. found only low levels of complement-mediated NF-κB activation upon infection with HAstV compared to adenovirus and human papillomavirus virus-like particles, suggesting that HAstV has strategies to evade detection by complement factor C3 [72]. Tam et al. also determined that intracellular sensing of C3 activated the interferon regulatory factor 3 transcription pathways, resulting in a robust secretion of IFN-β [72]. HAstV ability to evade detection by C3, which is involved in triggering an interferon response, may be why Guix et al. and Marvin et al. detected low levels of type I IFN at later times post infection. Future studies involving HAstV mutants that do not bind complement molecules will delineate the role(s) of suppression of complement activation during astrovirus infection.

Studies in turkey poults suggest that astrovirus infection suppresses the immune system, making the host more susceptible to subsequent infections [60,76]. Turkey macrophage infection in vitro reduced macrophage viability and intracellular killing of a subsequent *Escherichia coli* (*E. coli*) challenge compared to uninfected macrophages [60]. In vivo experiments showed that macrophages isolated from TAstV-infected poults had a percentage of phagocytic macrophages compared to macrophages isolated from uninfected poults [60]. These macrophages had less interleukin (IL)-1 and IL-6 activity and had fewer *E. coli* per macrophage compared to macrophages isolated from uninfected poults [60]. TAstV-infected poults also recruited almost half the number of Sephadex-elicited inflammatory cells to the abdominal cavity compared to uninfected poults [60]. These results correlate with an increased number of *E. coli* in the spleens of TAstV-infected poults [60], suggesting that TAstV infection decreases macrophage viability and function, rendering the host more susceptible to secondary bacterial, and possibly viral, infections.

## 6. Conclusions

In summary, although our understanding of the immune responses to astrovirus infection has progressed over the years, further studies are required to fully characterize the cellular and molecular factors that mediate the immune response to astrovirus infection, including identifying the correlates of protection that inhibit viral replication and pathogenesis. Future areas of inquiry include the absence of a potent inflammatory response following the rupture of endosomal membranes [12], as seen with other viral-induced membrane ruptures [77,78] and determination of how astrovirus particles exit cells through a non-lytic mechanism [21]. Additionally, an improved understanding of the mechanisms underlying viral inhibition of the inflammatory response and how type I IFN limits astrovirus-induced barrier permeability are critical for the development of therapeutics and treatment protocols. The development of a mouse model will be invaluable in understanding the viral replication kinetics and innate and adaptive immune responses to astrovirus infection in mammals. A full understanding of the immune response to astrovirus infection is necessary if we are to control and prevent astrovirus diseases.

## Figures and Tables

**Table 1:** Astrovirus localization in extra-intestinal tissue.

Animal/Human	Tissue(s)	Method(s) of Detection	Reference
Turkey	Bursa, Thymus, Spleen, Kidney, Liver, Skeletal Muscle, Bone Marrow, Pancreas, Plasma	RT-PCR, immunofluorescence, infectious virus isolation	[6,23]
Duck	Liver	RT-PCR	[30]
Cow	Brain	RT-PCR, Sequencing	[27,28,29]
Mink	Brain	Sequencing	[26]
Pig	Blood	RT-PCR, Sequencing	[31]
Human	Blood	RT-PCR, Sequencing	[32,33,34]
Human	Cerebrospinal Fluid	Sequencing	[34]
Human	Urine	Sequencing	[34]
Human	Brain	Sequencing, Immunohistochemistry	[35,36,37]
Human	Nasopharyngeal swab	RT-PCR, Sequencing	[32]
Human	Pharyngeal swab	RT-PCR, Sequencing	[32,38]
